# Do Shared Digital Workspaces Boost Integration? The Case of One Early Intervention Initiative for Vulnerable Children in Norway

**DOI:** 10.5334/ijic.5710

**Published:** 2022-05-13

**Authors:** Marit Kristine Helgesen, Helge Ramsdal

**Affiliations:** 1 Østfold University College, NO

**Keywords:** digital workspace, integrated care, cross-sectoral, collaboration

## Abstract

**Introduction:**

The paper discusses the implementation of a digital workspace to facilitate collaboration in health and social services for vulnerable children and adolescents in eight Norwegian municipalities. The purpose of the workspace is to enhance collaboration independent of space and time. Collaborating services are schools, kindergartens, school health services, educational services and child welfare services.

**Methods:**

The data analysed are from semi-structured interviews with project leaders in primary care, responses of primary care professionals to open questions in a survey, and results from two questions in three subsequent surveys.

**Results:**

Project leaders held great expectations of increased collaboration. Variations were found regarding how far the implementation of a new workspace precluded previous methods of collaboration and whether retaining a familiar workspace necessitated strengthening resources to negotiate using the workspace. Organisational and professional cultures hindered the implementation of the workspace.

**Discussion:**

Interrelated barriers to collaboration were found at the professional, organisational and systemic levels. Some professionals could adapt the workspace to their existing tasks while others could not. Primary care providers need to strengthen their organisations while implementing the workspace.

**Conclusion:**

Concerted action at national and municipal level is needed to successfully implement digital tools.

## Introduction

Health and wellbeing for children has increasingly been set on the agenda internationally, as well as *both nationally and locally* in Norway [1,2,3]. Recently, Norway has witnessed serious cases in which children have faced vulnerable life situations – and even death – as a result of parental drug or alcohol abuse, neglect, sexual abuse and violence. Recent analyses concluded that crises arose from coordination problems: when vulnerable children needed integrated care involving horizontal as well as vertical coordination, it could not be provided [4,5]. Care organisations are complex, and decentralised services and distributed responsibility make integrated care work one of the most difficult challenges to health and social care [3,6,7].

Norwegian authorities have initiated a diverse set of measures to prevent and reduce severe events involving children and to promote their health and wellbeing [4,5,7,8]. Providing services for children in need in Norway is complex. Care and support take place across schools, kindergartens, primary physical and mental health services, social services, child welfare and school health services [7,9]. All these services are municipal responsibilities in Norway, while secondary health services (hospital care) are the responsibility of the health enterprises. Therefore, the aim of this paper is to present and discuss one early intervention initiative to strengthen municipalities’ cross-sectoral work for vulnerable children.

The early intervention initiative in question was developed by the Norwegian Health Directorate, eight municipalities and their project leaders. Six out of eight project leaders were organisationally affiliated with the municipal administration, the last two with the school health and welfare services. A template for the intervention was created containing three key elements:

1. A log which initially was not elaborated as a digital shared workspace became so when municipalities started their work. The log was to document measures implemented for enrolled children and efforts at coordination, ensuring that information was passed on and that discontinuities in care provision were avoided [8]. While the suggestion was to use a standard word processing tool to establish logs, three municipalities started using a digital platform already known to them, which gave few opportunities for registering information. The other five municipalities implemented a newly developed digital workspace. Text fields for information on interventions, feedback on the outcomes and comments from professionals were included. This turned out to be a complex information system [10].
2. Detailed guidelines provided additional capabilities for registering information about children and instructions on which interventions should be implemented first by the service raising the concerns. This is the only instance where the “responsible organisation” is mentioned in the guidelines. If several services need to integrate their work, then planning, deciding and implementing interventions across these services should be documented.
3. A coordinator, a professional working for the service unit first raising concerns, should be appointed. This is the only instance a “responsible professional” is mentioned. The coordinator should be specifically appointed to take responsibility for establishing and maintaining the log, involving children and guardians and being their contact person when dealing with the involved services [8].

We present and discuss the implementation of the log. Some municipalities introduced work-place training for all employees involved, others for designated professionals while some provided information only.

## Theoretical approach

Integration can be achieved as a combination of top-down structural coordination on the one hand and process-oriented, bottom-up collaborative activities on the other [11,12]. Characteristics such as common goals, shared responsibility and practice define collaboration [11]. Efficient integrated care is not possible without communication and information, and digital information systems allow more services to be combined to build networks of independent organisations [13]. It is normatively possible, to define integrated care as the “delivery of coordinated care [that] requires integrated organisational networks that collaborate; and effectively and efficiently transfer synchronised information and manage resources” [14, p. 625]. Attaining such goals needs to be facilitated by clear governance arrangements [15], and a digital workspace consciously designed to promote integration can help providers in such an endeavour. Thus, information systems hold great promise for improving the quality of integrated care. Other key expectations towards implementing such systems are enhanced communication and information, possibilities to transcend time and space barriers, greater recognition of professionals’ complementary skills, roles and responsibilities, more efficient decision- making and implementation of interventions and more autonomous professionals [16,17,18]. Further, it holds promise for reducing costs and making possible innovative models of integrated care [13,19].

To achieve integrated care, information systems innovations can only come through transformations of the way organisations and professionals work [17]. Further, such information systems are in use in many countries and questions are raised as to whether they in fact enhances effectiveness and efficiency or if they impair rather than improve organisational efficiency [10]. Other critiques are that information systems are costly to purchase, implement and maintain and a source of frustration to practitioners. The critique further contains that systems make demands on street level bureaucrats’ work time and take them away from their core business of working with service users [16,20,21] and that workarounds are performed to adapt the systems to the organisation or to citizens seeking help [22,23]. Whether such systems function to enhance service integration and make services effective and efficient still is an open question and needs to be researched empirically.

In light of both expectations and promises as well as the critique against information systems, the research question for our presentation of this case is whether implementing this workspace made integrated care for vulnerable children effective and efficient. We have three sub-questions: a) What are the expectations towards the workspace held by project leaders? b) Does the workspace promote integration among services in the municipalities? c) Do the municipal organisational and professional cultures support the implementation of the workspace?

## Methods

The procedure for the eight municipalities to take part in the development project included agreeing that their work would be evaluated and to facilitate surveys. Initially the early intervention initiative was a developmental project. It lasted from 2012 until 2017.

### Overview of surveys

**Figure 1:**
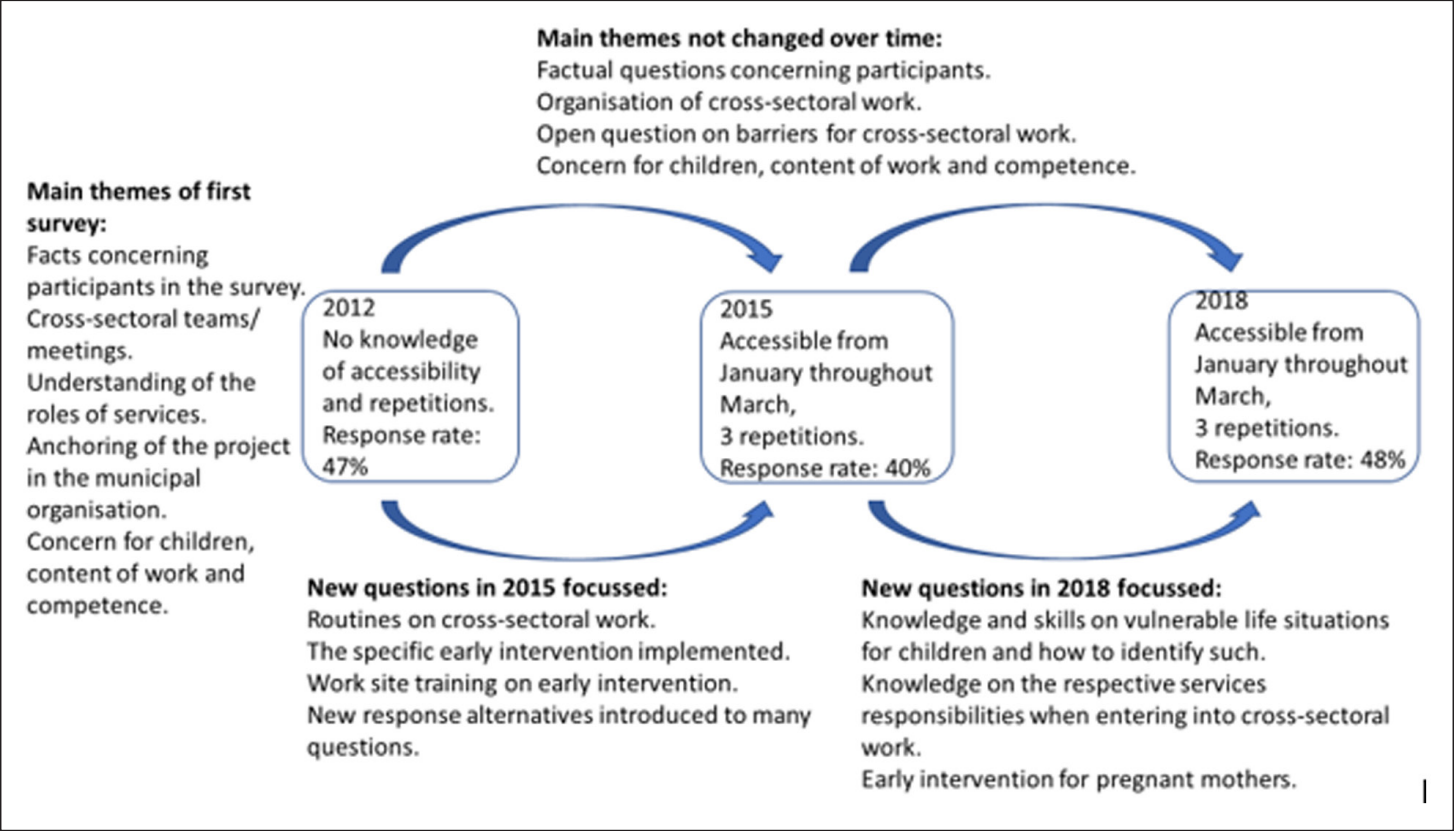
Overview of surveys.

Municipalities varied in size and were geographically dispersed. The first author analysed one ready-made dataset from a survey planned and conducted by the Health Directorate and took part in developing and analysing two ensuing surveys. All surveys were digital. Figure 1 below displays the survey process.

Surveys were sent to all employees of current services in all municipalities, and we received about 2000 responses each time. Table 1 displays numbers in more detail. In decreasing numbers, responses were received from schools, kindergartens, child welfare services, educational services and community and school health nurses. Schools are the services with the most employees.

**Table 1:** Surveys actually received by respondents, responses received, response rate and distribution on the categories’ practitioner/manager.


RESPONSES/YEAR	2012	2015	2018

Respondents receiving surveys	4782	4445	4155

Responses	2260	1765	2006

Response rate	47%	40%	48%

Practitioners	1808	1414	1683

Managers	452	351	323


No questions were asked about digital workspaces in the surveys. All had a number of free text answers and fields for free text comments. Open answers were given to the two questions cited in table four, and the last question: “Is there anything we have not asked about the developmental project and cross-sectoral work that you think we should know? If there is, please write it here”. Open answer questions are analysed for the 2018 survey only.

### Overview of interviews, themes and coding process

To gain more in-depth knowledge, the first author suggested conducting short interviews with the eight municipal project managers in 2012 and 2018. The first round of interviews concentrated on education while the second came to be preoccupied with digitalisation. Therefore, quotes from interviews are from 2018 only. Interviews lasted about 30 minutes and were transcribed and anonymised. This enabled a triangulation of methods [24]. Key questions from interviews, numbered 1–8, are presented in Table 2.

**Table 2:** Key interview questions.


How is the pilot organised in your local authority?

Which procedures have been established?

What is your opinion on involving the top administrative management, sector managers and service leaders in the pilot?

Do service leaders support this collaborative initiative?

What is your opinion on the willingness of the staff of this local authority to work across sectors in the pilot?


In analysing the interviews and comments on open questions in the survey, the stepwise deductive-inductive method was used [25]. It is based on six steps, and the three most relevant for this project were 1) the inductive identification of codes that could 2) be organised into themes and 3) to ensure that the most suitable concepts were deduced from the empirical data. Both authors read the interviews and questionnaire responses several times, grouped the identified codes that had a coherent content and decided which code groups, and how many, could be the main themes in a paper. The last step depended on the research problem identified, which in this paper was whether the digital shared workspaces enhanced effectiveness and efficiency in the integrative work. The coding and grouping process is presented in Table 3.

**Table 3:** Codes and themes in interviews and survey comments.


BASIC CODES IN INTERVIEWS: FACTORS INFLUENCING THE CROSS-SECTORAL IMPLEMENTATION OF THE DIGITAL WORKSPACE (INTERVIEWS CONTAINING THE CODE)	THEMES (GROUPS OF CODES)	RESEARCH QUESTION

Importance of implementing a digital workspace (all project leaders) Implementing a new workspace (local authorities nos. 1, 2, 3, 5, 7)	Expectations for the digital workspace	

Implementing known workspace (4, 6, 8)		

Existence of cross-sectoral teams/collaborative initiatives (1, 4, 8)		

Efficient and relevant meetings/Routines for meetings/procedures (1, 4, 5, 6, 7, 8)	Promoting not promoting integration	

Single purpose agencies (1, 7 + comments from the survey)		Digital workspace boosts integrative work

Professional resistance to implementing a digital workspace (1, 2, 3, 6, 8+ comments from the survey)		

Professional and organisational regulations constrain the use of a digital workspace (2, 5, 6, 8 + comments from the survey)	Professional and organisational culture	

Not establishing consensus on when to use the digital workspace (all local authorities)		


### Limitations

The design had been representative if the research project at the outset had had a clear mixed-methods or diachronic data collection design. Then, the data collection and results would have been strengthened concerning the validity and reliability of both surveys and interviews.

### Ethics

Consent for the survey was obtained if participants returned the questionnaire. For the interviews, consent was obtained orally as the interviews were conducted by phone. Information given is treated in line with the guidelines of The National Committee for Research Ethics in the Social Sciences and the Humanities.

## Results

### Expectations towards the digital workspace

As the methods section reveals, developing the workspace was initially neither included in the early intervention project nor in the evaluation. Therefore, in the analysis, respondents were not expected to appreciate the importance of the digital workspace when the interviews and the surveys were conducted. Nevertheless, they did. All interviewees mentioned the digital workspace, the necessity of having one and they assumed it promoted coordination and collaboration. The quotation below is representative:

“The workspace ensures that interventions for a child or adolescent can be implemented simultaneously. Interventions are not sequential. Every actor involved can see the interventions provided and their effect. Other actors may view cases differently and consider them on the basis of their particular professional viewpoint. A child or adolescent can theoretically receive measures from all support services simultaneously. Everything is visible in the workspace”.

Expectations were high as to the collaborative strength of the workspace and possibilities for negotiations on early intervention initiatives. A comment in the survey confirms this:

“I see only advantages in that participants can communicate in the workspace in between meetings. In meetings, we can project goals and measures on a screen and everybody can see. We can evaluate and change measures.”

Five municipalities implemented a newly developed workspace while three decided to use one they had experience with. The project leader in municipality 2 talked about how the workspace was an important novelty and therefore anticipated enhancing integration efforts:

“The digital workspace is the novelty […] that is the change. It is easier for more service providers to work simultaneously. Without a workspace for collaboration, the required communication with local care services would have been a lot more difficult. The workspace is important […].”

In municipalities that implemented the familiar workspace, leaders spoke of challenges with the workspace functionalities, as in this example from municipality 6:

“[Our workspace] is difficult to use and we discuss how to do this. You can do almost anything because it has few barriers and it can obviously be used across service providing units, and that is a challenge as well.”

There were few restrictions as to who could log on and few designated fields for storing information. Actors involved had to discuss procedures and thus had to maintain an arena for negotiating integration.

Implementing the digital workspace was a conscious decision in all municipalities, whether the workspace was new or familiar. Expectations for it to improve integration and collaboration were high.

### Does the workspace promote integration?

Cross-sectoral integration to promote children’s health and wellbeing requires services to be collaborative. The actors need to negotiate concerns and interventions. The workspace provides such possibilities, and the meetings arranged in addition to the workspace function as resources facilitating communication and negotiation. Managers and professionals’ knowledge of integrative initiatives (meetings and teams) indicate whether these are in focus. Table 4 shows a decrease from 2012 to 2018 in knowledge of meetings and teams held by professionals and managers in all services, except for kindergartens and school health services. In 2015, fewer survey responses were returned, and the decrease is therefore most significant between 2012 and 2015, after which the figures increase again. Results for the second question show a decrease for most alternatives during the years 2012–2018, but an increase for “parents and guardians are invited to meetings on cases in which they are partners”. The decrease for “I know how to put cases on the cross-sectoral team agenda” and “I know who is responsible for arranging the cross-sectoral meetings” suggests that the resource cross-sectoral integrative forums were discontinued.

**Table 4:** Key themes and results from the surveys. Percent.


QUESTION	RESULTS IN PERCENTAGES, MULTIPLE ALTERNATIVES	2012	2015	2018

Which services are represented in teams or other types of collaborative initiatives?	Educational services	87	60	80

	Child welfare	84	59	68

	Health centre	53	42	50

	Schools	62	47	49

	Kindergartens	38	41	45

	School health services	40	32	42

Do you have knowledge of the collaborative initiatives in this local authority?	I know how to put cases on the cross- sectoral team agenda	80	55	36

	I know who is responsible for arranging the cross-sectoral meetings	64	45	42

	We have procedures for information exchange related to cross-sectoral meetings	45.5	41	32

	Parents and guardians are invited to meetings on cases in which they are partners	22	44	33


A comment from the survey mentions this change:

“[…] previously we had a team for consultations in which different services were present. It was terminated. At school we still have the psycho-social team […].”

Two interviewed project leaders mentioned teams for integration in special needs education. Such teams are closely connected to schools, which indicates that schools operate independently in this field.

Some interviewees stated that municipal services had cross-sectoral meetings as well as a workspace:

“We have created a system where we decide which individual cases to handle in the workspace, and based on those we interact there. We continue our established routines for cross-sectoral meetings to discuss the challenges we have.” (Municipality 7)

Others argued that they changed their meeting arrangements to become more efficient. The project leader in local authority 5 clarified this:

“The aim is to have efficient meetings and discuss goals, measures and evaluations. We spent meeting time discussing things that are of limited benefit.”

This indicates that service providers in some municipalities, presumably those who implemented the new workspace, shifted their focus from physical meetings and team discussions to using the digital workspace as their only collaborative arena, while those who still held physical meetings in addition to using the workspace improved the meetings to become more efficient.

### Organisational and professional culture

Organisational and professional culture affects attitudes towards taking part in integrative initiatives. We found some distinctions in attitudes across professions and services. Nurses working in health centres and school health services expressed positive attitudes. One survey respondent commented:

“In the health centre we are obliged to write patient records and quality in our work is important to us. So, we welcome […] the workspace.”

Nevertheless, nurses found that they were responsible for establishing and maintaining logs. Another comment was:

“I find that schools sometimes do not want to coordinate. They want the school nurses to do it. I find that teachers do not know what the workspace is.”

A respondent from a child welfare agency made a similar comment:

“It would be natural to establish logs in many cases that involve child welfare, or to start them right after child welfare starts its work. Concerns [about children’s health and wellbeing] have been raised and measures implemented before child welfare is involved. In practice, there is often no log in child welfare cases.”

These quotes indicate that school nurses and child welfare professionals became responsible for initiating collaboration and maintaining digital communication. They realised that they knew more about the digital workspace and how to use it than teachers in schools and kindergartens. Hence, they expressed concerns that the resistance found in schools hindered efficient decisions and implementation of interventions. Teachers’ attitudes proved these concerns to be justified, as one survey comment shows:

“For the schools, the system is just a new digital tool and there’s little motivation for using it. Increased digitalisation has led to some extra work for schools in general.”

Other teachers commented that they did not want to use the workspace; they found it to be inefficient and cumbersome and in competition with their specific educational tools. From their point of view, the workspace had a health focus and enabled the health services to work with a clear and straightforward system. Project leaders stated that use of the workspace was challenged by school procedures. The project leader in municipality 7 said:

“For instance, they [the schools] report challenging operational situations, […]. This depends on practical facilitation and technical skills.”

The interviewee in municipality 8 clarifies this:

“Teachers used to be autonomous […]. That culture is changing but it is still strong.”

Many of the schools involved did not use digital tools. The staff were therefore neither involved in the development of the initiative nor in using the workspaces. This indicates that service managers and professional groups need to make efforts to change their cultures towards integration and understanding of the grey zones between services and professions.

## Discussion

The paper’s research question was whether a shared workspace could make integrated services for children and adolescents more effective and efficient. Three sub-questions were posed. Concerning the first, what the expectations held by the project leaders towards the workspace were, the analysis showed that they were high. They believed it to facilitate integration articulated as simultaneous implementation of interventions, shared goals, communication of information and negotiating of interventions as well as an improved evaluation of integrated interventions. Further, they believed it to enhance collaboration, thus making integration more effective and efficient. As the result presentation reveals, neither common goals nor shared responsibility and practice were features characterising the way interviewees and respondents talked about the workspace. On the contrary, results showed a lack of commitment [11].

Especially teachers argued that they did not have common ground with the other services and that they wanted to work with the teams and digital tools specific for schools. They did not trust the actual workspace to accommodate the needs of schools and held on to their relative autonomy [12], not using the workspace for communicating and sharing information. While all professional groups represented in the empirical analysis experienced frustrations concerning the workspace [15], teachers in particular articulated theirs and they probably thought that working with it made demands on their work time and took them away from teaching; their core task [21]. School nurses were left with the responsibility of establishing and maintaining logs, while the child welfare agency experienced frustration over not being included. As schools and kindergartens are important services, which see and talk to children and adolescents every day, teachers’ withdrawal from collaboration is challenging. When early intervention is not successful, concerns for children become future cases for the child welfare agency. Hence, the expectancies held by project leaders that the workspace would enhance effectiveness and efficiency in integration were not found among the professionals. This is in line with Gillingham’s [10] findings from Australia that complex information systems took up too much time from professionals working with children and youth.

Concerning the second sub-question, whether the workspace promoted integration we found that the service units and professionals merely co-existed. Collaboration neither increased, as teachers did not trust the workspace and withdrew from working with it, nor was top-down coordination initiated. When taking a closer look at differences between municipalities that implemented the complex workspace and those who retained the simpler one, the result on collaboration can be modified. While the latter kept their cross-sectoral meetings, which were held in addition to using the workspace, to evaluate interventions, those implementing the complex workspace discontinued meetings and teams. It is known to front line professionals that talk is effective [22], and the performed empirical analysis showed that meetings were a valuable resource and should be embedded in the technology to facilitate service integration. The need for physical meeting places – time and space – is constraining on service integration and the complex workspace had text fields for including specific information, a feature that could elevate the collaboration from the time and space barriers [20]. As is shown in research from Belgium [22], such text fields may constitute social workers data collection and does not facilitate talk. As well research from Denmark argues that technology may constrain service provision and lead professionals’ to take up the practice of workaround to escape the constraints or to adapt technology to the actual service provided [22]. Whether teachers’ withdrawal is a workaround can be questioned, but it probably is a result of believing that resources can be used otherwise to enhance services for school children. Nevertheless, the feature of elevating the collaboration from the time and space barriers probably was believed by project leaders to enhance efficiency; however, it did not enable an awareness towards interdependency among service units and professionals.

The third sub-question was whether the municipal organisation and professional cultures supported the implementation of the workspace. The above discussion shows they did not. However, the empirical analysis shows that professionals differ in their appraisal of the workspace. School nurses and child welfare workers approached the workspace more or less positively, acknowledging the interdependency necessary to accentuate the grey zones between professionals. Establishing and maintaining logs was in line with the work tasks of school nurses, even though they seemed to be somewhat reluctant to take on a new work task. Child welfare workers experienced the absence of information as a system failure. This could have been avoided if logs had been established and maintained before cases were handed over to the child welfare agency.

Teachers on their part, approached this early intervention initiative rather negatively, not being willing to accept taking on new work tasks. As is shown by Barr et al [16] technologies may challenge professional spheres of influence and the teachers in our study may have felt threatened as they experienced that the workspace did not align with their work tasks and their organisational culture. Thus, implementing the workspace did not promote the recognition of professionals’ complementary skills, roles and responsibilities assessed necessary for collaboration and new ways of working to take place [17]. At the organisational level, this can be analysed as schools not recognizing other municipal service providing units as part of their task environment; the features of the environment relevant to the schools’ supply of inputs, dispositions of outputs and the power-dependence relations within which the organisation conducts its exchanges [26 p. 125]. On the contrary, schools established intra-sectoral teams to enhance children’s health and wellbeing. They remained autonomous [12], and independent teachers were unwilling to enter into networks by using the workspace to collaborate.

As is discussed, implementing a digital workspace is not a panacea to integrate services. Systemic, organisational and professional barriers to collaboration and coordination are manifold [9,11,15]. Among the organisational barriers are administrative support in the form of leadership and management. Leaders must convey the vision that the workspace is the new collaborative practice, motivate professionals to use it and create an organisational culture in which this is possible. Management needs to steer the distribution of resources, such as teams, training, support from dedicated IT-staff, or other organisational requirements. At the systemic level, professionalization is characterised by domination, autonomy and control [27], and these components thus infuse the organisations with values [28], forming different organisational cultures in service providing units.

These barriers are at different levels and interrelated, thus constituting the institutional environment determining the path professionals, leaders and managers follow in making decisions on integration. Some patterns of behaviour are seen as more appropriate than others [29]. When the regulations, norms and cognitive understandings brought by the workspace did not resonate well with schools, it was because teachers and schools make decisions and act within the institutionalised environments in which they are embedded. Agreeing to use the workspace was not considered appropriate due to the extra workload involved or because the workspace was defined as a tool made to accommodate the needs of the health sector.

To ensure stakeholder acceptance, institutional rules and norms considered appropriate for all the different professional groups involved must be established by concerted actions from national and municipal governments. Firstly, the expectations that digitalisation would lay the foundations for increased value creation, innovation and increased productivity [19] did not result in any excess capacity in municipal services. Implementing digital tools is costly for local government, and national funds need to be generous. Secondly, a study of the overall task environment in which the workspace will function would enable identification of interdependencies among service providing units and professional grey zones in which resistance is initiated. Such endeavour can reduce tensions among stakeholders [11], change the actors’ cognitive structures and make them more positive towards the workspace. Their experience would not only consist of receiving an extra workload by implementing cumbersome digital procedures. A key goal should be to ensure that the professionals involved become positive advocates of the digital tool, and management should communicate how professions and technology are intertwined and how using the technology may change situations for children and professional tasks, as well as the work and organisational environments of each service.

## Conclusion

Horizontal integration of the services involved in improving the wellbeing of children was not boosted by implementing a digital workspace in municipal service providing units. Our study found that the workspace as such promoted neither top-down coordination nor bottom-up collaboration. It will probably continue this way until central and local government coordinate their digitalisation efforts providing services for vulnerable children.
